# Heat shock enhances outer-membrane vesicle release in *Bordetella* spp.

**DOI:** 10.1016/j.crmicr.2020.100009

**Published:** 2020-09-17

**Authors:** Eline F. de Jonge, Melanie D. Balhuizen, Ria van Boxtel, Jianjun Wu, Henk P. Haagsman, Jan Tommassen

**Affiliations:** a Section Molecular Microbiology, Department of Biology, Faculty of Science, Utrecht University, Padualaan 8, 3584 CH Utrecht, the Netherlands; b Institute of Biomembranes, Utrecht University, Utrecht, the Netherlands; c Section Molecular Host Defence, Division Infectious Diseases & Immunology, Department of Biomolecular Health Sciences, Faculty of Veterinary Medicine, Utrecht University, Utrecht, the Netherlands

## Abstract

Pertussis, also known as whooping cough, is caused by the Gram-negative bacterium *Bordetella pertussis*, an obligate human pathogen. Despite high vaccination rates in high-income countries, resurgence of pertussis cases is an occurring problem that urges the necessity of developing an improved vaccine. Likewise, the efficacy of vaccines for *Bordetella bronchiseptica*, which causes similar disease in pigs and companion animals, is debatable. A promising approach for novel vaccines is the use of outer membrane vesicles (OMVs). However, spontaneous OMV (sOMV) release by *Bordetella* spp. is too low for cost-effective vaccine production. Therefore, we investigated the influence of growth in various media commonly used for culturing *Bordetella* in the Bvg^+^, i.e. virulent, phase and of a heat shock applied to inactivate the cells on OMV production. Inactivation of the bacterial cells at 56 °C before OMV isolation greatly enhanced OMV release in both *Bordetella* spp. without causing significant cell lysis. The growth medium used barely affected the efficiency of OMV release but did affect the protein pattern of the OMVs. Differences were found to be related, at least in part, to different availability of the nutrient metals iron and zinc in the media and include expression of potentially relevant vaccine antigens, such as the receptors FauA and ZnuD. The protein content of OMVs released by heat shock was comparable to that of sOMVs as determined by SDS-PAGE and Western blot analysis, and their heat-modifiable electrophoretic mobility suggests that also protein conformation is unaffected. However, significant differences were noticed between the protein content of OMVs and that of a purified outer membrane fraction, with two major outer membrane proteins, porin OmpP and the peptidoglycan-associated RmpM, being underrepresented in the OMVs. Altogether, these results indicate that the application of a heat shock is potentially an important step in the development of cost-effective, OMV-based vaccines for both *Bordetella* spp.

## Highlights

• Heat inactivation of *Bordetella* stimulates outer membrane vesicle (hOMVs) release.
• hOMVs are released without extensive cell lysis.
• Protein composition of hOMVs is similar to that of spontaneous OMVs (sOMVs).
• Protein composition of hOMVs and sOMVs is deviant from that of the outer membrane.
• hOMVs are a promising tool for the development of novel *Bordetella* vaccines.

## Graphical abstract

**Figure:**
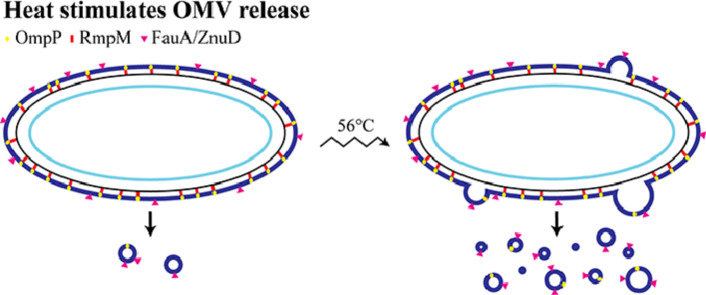

## 1 Introduction

*Bordetella pertussis* is a Gram-negative bacterium causing pertussis, also known as whooping cough (Mattoo and Cherry, 2005). It is a human-adapted species derived from its ancestor *Bordetella bronchiseptica*, which is associated with, amongst others, atrophic rhinitis in pigs and kennel cough in dogs (Gerlach et al., 2001)*.* In the first half of the 20th century, the first whole-cell pertussis (wP) vaccines were developed and introduced (Mattoo and Cherry, 2005). Although wP vaccines have been proven to be effective, reactogenicity turned out to be a major issue (Cody et al., 1981) and was shown to be related to the presence of endotoxin (Geurtsen et al., 2006). Therefore, new, acellular pertussis (aP) vaccines were developed, containing one to five purified antigens. Reactogenicity of these vaccines is decreased compared to wP vaccines, but pertussis has been resurging in the past two decades even in countries with high vaccination rates (Cherry, 2012; Mooi et al., 2014). This resurgence is due to genetic changes in circulating *B. pertussis* strains, rapid waning of immunity, and failure of aP vaccines to protect against *B. pertussis* colonization, amongst others (Mooi et al., 2014; Warfel et al., 2014). Current *B. bronchiseptica* vaccines are composed of whole cells, but the efficacy of these vaccines is debatable (Ellis, 2015). Thus, for both *B. pertussis* and *B. bronchiseptica*, there is a need for novel vaccines.

A promising new approach for vaccine development is the use of outer membrane vesicles (OMVs). OMVs are non-replicative blebs of 10–300 nm in size naturally released from the outer membrane (OM) of Gram-negative bacteria (Hozbor et al., 1999; Ellis and Kuehn, 2010; Schwechheimer and Kuehn, 2015). A challenge in the development of an OMV-based vaccine is the low production of spontaneous OMVs (sOMVs) by *Bordetella* species (Hozbor et al., 1999). OMV production in various Gram-negative bacteria is influenced by environmental stresses, such as temperature and antibiotics (McMahon et al., 2012; MacDonald and Kuehn, 2013), and cellular stresses, e.g. periplasmic stress (McBroom and Kuehn, 2007; MacDonald and Kuehn, 2013). Periplasmic stress can be caused by accumulation of peptidoglycan (PG) fragments, lipopolysaccharide (LPS) or misfolded proteins in the periplasm and has been shown to increase vesiculation in *Escherichia coli* (McBroom and Kuehn, 2007; Schwechheimer et al., 2014). The increased vesiculation by *E. coli* at higher temperatures is probably also due to the accumulation of misfolded proteins, which the bacteria may shed by their inclusion in OMVs (McBroom and Kuehn, 2007). Furthermore, the composition of the growth medium can influence OMV production, as has been shown in *Francisella novicida* and *Neisseria meningitidis* (Pierson et al., 2011; Santos et al., 2012; Sampath et al., 2018).

In this study, we investigated the influence of medium composition and heat shock on OMV release, focusing on *B. pertussis* and *B. bronchiseptica* as a one-health approach. Various media have been described for the growth of *B. pertussis*. The first serum-free liquid medium for Bvg^+^, i.e. virulent-phase, *B. pertussis* was described by Hornibrook (Hornibrook, 1939). The main component of Hornibrook medium is hydrolyzed casein (casamino acids) as a nitrogen and carbon source, and the medium further consists of inorganic salts, starch, and either glutathione or cystine as a sulfur source. In this medium, an alkaline reaction takes place limiting *B. pertussis* growth. The alkaline reaction is prevented in Verwey medium by increased phosphate concentrations (Verwey et al., 1949). In a further optimized medium, the Stainer-Scholte (SS) medium, which is widely used for the growth of *Bordetella*, the casamino acids are replaced by proline, cystine, and glutamic acid (Stainer and Scholte, 1971). In addition, starch was omitted as *B. pertussis* cannot use it as a carbon or energy source because it does not possess a functional glycolysis (Stainer and Scholte, 1971; Thalen et al., 1999). In all media mentioned above, *B. pertussis* grows on amino acids, resulting in the accumulation of ammonium due to an imbalance between carbon and nitrogen availability (Thalen et al., 1999). Ammonium accumulation can be prevented by the addition of an extra carbon source, such as lactate. The addition of lactate resulted in a balanced medium, the Thalen-IJssel (THIJS) medium (Thalen et al., 1999). We determined OMV release by *B. pertussis* and *B. bronchiseptica* in three different media, namely Verwey, SS and THIJS medium. In addition, the effect of a heat shock, which is often used to inactivate the bacteria, on OMV release and on the quality of the OMVs was assessed.

## 2 Materials and methods

### 2.1 Bacterial strains and growth conditions

*B. pertussis* strain B213, a streptomycin-resistant (Sm^R^) derivative of Tohama I (King et al., 2001), and *B. bronchiseptica* strain BB-D09-SR, a spontaneous Sm^R^ derivative of strain BB-D09 isolated from dog (isolate number 2,170,524,052; Veterinary Microbiological Diagnostic Center, Division Infectious Diseases & Immunology, Faculty of Veterinary Medicine, Utrecht University), were grown on Bordet-Gengou (BG) agar (Difco) plates supplemented with 15% (v/v) defibrinated sheep blood (bioTRADING) at 35 °C. For liquid cultures, bacteria were scraped from BG plates and pre-grown for two days in Verwey medium at 35 °C while shaking at 175 rpm. Cells were harvested, washed with physiological salt solution, and diluted to an optical density at 600 nm (OD_600_) of 0.05–0.1 in Verwey, SS, or THIJS medium (Table S1, which is provided in supplementary materials). When indicated, media were supplemented with 1 g/L of heptakis (2,6-di-O-methyl)-β-cyclodextrin (heptakis) (Sigma-Aldrich) to protect *B. pertussis* against fatty acids that are produced during growth (Frohlich et al., 1996). In some experiments, starch was omitted from the Verwey medium as indicated. Subsequent growth at 35 °C was monitored by measuring the OD_600_ with a Novaspec III+ spectrophotometer (Biochrom). To kill bacterial cells, cultures were incubated for 1 h (unless otherwise notified) at 56 °C in a water bath. Viability of the cells was determined by spotting 10 µL of 10-fold serial dilutions of the cultures in physiological salt solution on BG agar plates. *E. coli* strains DH5α, BL21(DE3) and MG1655 were grown at 37 °C on lysogeny broth (LB) agar plates or in liquid LB while shaking at 200 rpm. For selection or plasmid maintenance, 100 µg/mL of ampicillin was added to the medium.

### 2.2 OMV isolation and quantification

Bacterial cultures were grown for one day in conical tubes with an air:liquid ratio of 3.33:1 or, alternatively, for two days in baffled flasks with an air:liquid ratio of 5:1. Subsequently, cultures were incubated or not at 56 °C, as indicated. Bacterial cells were pelleted by centrifugation at 5000 x *g* for 10 min, and supernatants were passed through 0.45-µm pore-size filters (Sarstedt). In the case of 100-mL cultures, cell-free filtrates were concentrated using 100-kDa cutoff centrifugal filter units (Amicon). OMVs were pelleted by ultracentrifugation for 2 h at 40,000 rpm and 4 °C (Beckman Coulter Optima LE-80 K, Type 70 Ti rotor), and resuspended in phosphate-buffered saline (PBS) or 2 mM Tris–HCl (pH 7.5).

OMVs were quantified based on protein content using a bicinchoninic acid (BCA) assay (Pierce) or a Lowry DC protein assay (Bio-Rad) according to the manufacturers’ instructions or based on lipopolysaccharide (LPS) content using the purpald assay (Lee and Tsai, 1999). For the latter assay, 50 µL of OMVs in PBS were mixed with 50 µL of 32 mM sodium periodate and incubated for 25 min at room temperature (RT). Then, 50 µL of 136 mM purpald (Sigma) in 2 M NaOH were added and incubated for 20 min at RT, and the reaction was stopped by the addition of 50 µL of 64 mM sodium periodate for 20 min at RT. Subsequently, 20 µL of isopropanol was added to eliminate foam. Absorbance at 550 nm was measured and known concentrations of 2-keto-3-deoxyoctonate ammonium salt (KDO, Sigma) were used to plot a standard curve.

### 2.3 Separation of inner membrane (IM) and OM by sucrose density gradient centrifugation

*B. pertussis* was grown for two days in SS medium and subsequently inactivated by incubation for 30 min at 56 °C. In the case of *E. coli*, an overnight culture of strain MG1655 was diluted 1:50 in fresh LB and grown for 3 h, after which the cells were either inactivated by incubation for 30 min at 56 °C or incubated for 30 min on ice. Cells were harvested by centrifugation at 10,000 rpm (Eppendorf 5920-R centrifuge, FA-6 × 50 rotor) for 10 min and washed with physiological salt solution. Spheroplasts were made as previously described (Osborn et al., 1972). Briefly, cells were resuspended to an OD_600_ of 30 in 7 ml of 0.75 M sucrose, 10 mM Tris–HCl (pH 7.8). Then, 35 µL of 40 mg/mL lysozyme were added, followed by 14 mL of 1.5 mM EDTA (pH 7.5). The suspension was incubated for 30 min at RT. Spheroplasts were frozen at −80 °C, thawed, and 20 µg/mL of DNase and of RNase were added. Subsequently, spheroplasts were lysed by ultrasonication. Unbroken cells were removed via centrifugation for 10 min at 10,000 rpm for *B. pertussis* or at 2700 rpm for *E. coli* in an Eppendorf 5920-R centrifuge (FA-6 × 50 rotor). The supernatant was then centrifuged for 90 min at 40,000 rpm (Beckman Coulter Optima LE-80 K, Type 70 Ti rotor), and the resulting pellet was resuspended in 25 mM Tris–HCl, 1.25 mM EDTA (pH 7.5). Cell envelopes were loaded onto a discontinuous sucrose gradient consisting of a 3-mL cushion of 55%, four 2.5-mL layers of 50, 45, 40, and 35%, and a 1.8-mL top layer of 30% (w/w) sucrose, all in 25 mM Tris–HCl, 1.25 mM EDTA (pH 7.5). The sucrose gradient was centrifuged for 16 h at 25,000 rpm (Beckman Coulter Optima LE-80 K, SW28.1 rotor) at 4 °C and fractions were collected. Lactate dehydrogenase activity in the gradient fractions was determined as described (Osborn et al., 1972).

### 2.4 Antisera

Monoclonal antibodies directed against pertactin (Prn) and mouse antiserum directed against BrkA were kindly provided by Nathalie Devos (GlaxoSmithKline Biologicals SA), and rabbit antisera directed against GroEL and against SecA were from our laboratory stocks.

To obtain antisera directed against the major porin OmpP (BP0840), and the TonB-dependent receptors BP3077 (ZnuD) and FauA, the corresponding genes were amplified without their signal sequence-encoding parts by PCR from chromosomal DNA of *B. pertussis* strain B213 using the primers listed in Table S2. Amplicons were cloned into pET16b (Novagen) after NdeI/BamHI restriction digestion, and the resulting plasmids were introduced in *E. coli* BL21(DE3). Strains containing the pET16b derivates were grown to an OD_600_ of 0.6, after which 0.5 (OmpP and ZnuD) or 1 mM (FauA) isopropyl β-d-1-thiogalactopyranoside was added, and gene expression was induced for 2 h. The recombinant proteins, containing an N-terminal six-His-tag, accumulated in inclusion bodies. Harvested cells were washed twice in 10 mM Tris–HCl (pH 8) and disrupted by sonication. Inclusion bodies were pelleted by centrifugation (10 min, 2000 x *g*, 4 °C) and solubilized in 8 M urea, 100 mM glycine, 20 mM Tris–HCl (pH 8). Residual membrane fragments were pelleted by ultracentrifugation for 1 h at 100,000 x *g*, 4 °C. His-tagged proteins were purified by binding to Ni-NTA agarose beads (Qiagen), washed with a buffer containing 100 mM NaH_2_PO_4_, 10 mM Tris–HCl, 8 M urea, 20 mM imidazole, pH 8, and eluted with the same buffer containing 300 mM imidazole. Purified proteins were used to immunize rabbits at Eurogentec (Liège, Belgium).

To obtain antisera directed against the IM protein FtsH (BP1077) and OM protein (OMP) RmpM (BP0943), peptides CLPETDRYSMDKERL and CASNKTREGRAQNRR, respectively, were designed with the OptimumAntigen design tool and used to immunize rabbits at GenScript (Piscataway, New Jersey, USA). The N-terminal cysteine in both peptides was introduced for coupling to keyhole limpet hemocyanin as a carrier protein for immunization.

### 2.5 Sodium dodecyl sulfate-polyacrylamide gel electrophoresis (SDS-PAGE) and Western blotting

Bacterial cells, harvested by low-speed centrifugation, or isolated OMVs were mixed with sample buffer (Laemmli, 1970). Proteins were separated on 10 or 14% polyacrylamide gels by standard SDS-PAGE or by semi-native SDS-PAGE (Grijpstra et al., 2013). Gels were stained with Bradford reagent as described previously (Bos et al., 2015), with protein silver staining (Pierce) following manufacturer's instructions, or LPS silver staining (Tsai and Frasch, 1982). Alternatively, the separated proteins were transferred to a 0.45-µm pore-size nitrocellulose membrane (GE Healthcare). For immunodetection, the primary antibodies described above and horseradish peroxidase-conjugated goat anti-mouse or anti-rabbit IgG antisera (ThermoFisher) were used. As the anti-OmpP antiserum, which was raised against the denatured monomeric form of OmpP, does not recognize the natively folded oligomeric form of the protein, semi-native SDS-PAGE gels were heated under steam (Brok et al., 1995) to denature the folded protein *in situ* prior to blotting. Membranes were developed with the Clarity Western ECL Blotting Substrate (Bio-Rad). The intensity of band signals was determined using Image Lab v5.2.1 (Bio-Rad).

### 2.6 Detection of siderophore production

Siderophores secreted by bacteria were detected by the chrome azurol S assay (Schwyn and Neilands, 1987). Supernatants of bacterial cultures were mixed 1:1 with chrome azurol S solution and incubated for 1 h at RT, after which absorbance at 630 nm was measured.

### 2.7 Transmission electron microscopy (TEM)

Bacterial cells and OMVs were adsorbed to Formvar/carbon-coated copper grids for 10 min and washed three times with PBS. They were then fixed on the grids with 1% (v/v) glutaraldehyde in PBS for 10 min, washed twice with PBS and subsequently four times with Ultrapure water (Milli-Q). Samples were negatively stained by shortly rinsing the grids and subsequently incubating the grids for 5 min with methylcellulose/uranyl acetate (pH 4) on ice. Excess liquid was removed, and grids were air-dried. Samples were imaged using a FEI Tecnai 12 transmission electron microscope. OMV diameters were determined using ImageJ software.

## 3 Results

### 3.1 OMV release by *B. pertussis* and *B. bronchiseptica* in different growth media

**Fig. 1:**
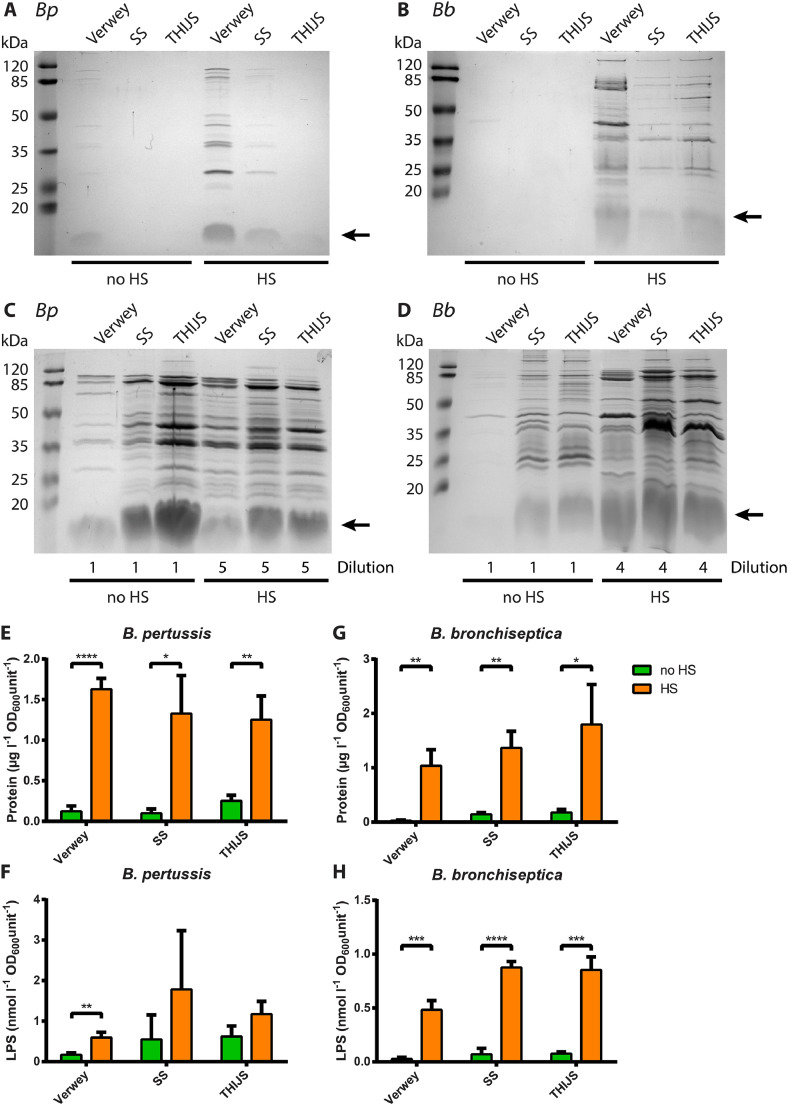
Influence of medium and heat shock on OMV release. *B. pertussis* (*Bp*) and *B. bronchiseptica* (*Bb*) were grown in Verwey, SS or THIJS medium and either killed by heat shock (HS) or not (no HS) at 56 °C for 1 h before centrifugation. OMVs were isolated from the supernatants of equal amounts of cells, based on OD_600_, from *B. pertussis* (**A**) or *B. bronchiseptica* (**B**) and analyzed by SDS-PAGE. OMV production was scaled-up by growing larger cultures of *B. pertussis* (**C**) or *B. bronchiseptica* (**D**) in baffled flasks with an air:liquid ratio of 5:1 for two days. In these experiments, the Verwey medium was not supplemented with starch to allow for quantification of the results. For growth of *B. pertussis*, the media were supplemented with heptakis. For SDS-PAGE analysis, hOMVs were four- (*B. bronchiseptica*) or five-fold (*B. pertussis*) diluted relative to sOMVs as indicated below the lanes. In panels **A-D**, LPS (lipid A plus core moiety), which is also stained with the Bradford reagent, is indicated with an arrow at the right and molecular weight markers are shown at the left. Protein content in *B. pertussis* (**E**) and *B. bronchiseptica* (**G**) OMVs was quantified using a Lowry assay. Values are depicted as the amount of protein per liter of bacterial culture per OD_600_ unit. LPS content in *B. pertussis* (**F**) and *B. bronchiseptica* (**H**) OMVs was quantified using the purpald assay. Values are depicted as the amount of LPS per liter of bacterial culture per OD_600_ unit. (**E-H**) Bars represent mean values with standard deviations of three biological replicates. Significant differences were determined with multiple *t* tests using GraphPad Prism 6 and are indicated by asterisks (*, *p* ≤ 0.05; **, *p* ≤ 0.01; ***, *p* ≤ 0.001; ****, *p* ≤ 0.0001).

To determine if growth-medium composition or a heat shock influence OMV release, *B. pertussis* strain B213 and *B. bronchiseptica* strain BB-D09-SR were grown in Verwey, SS or THIJS medium. It is noteworthy that, although SS and THIJS medium were successively developed as optimized media compared to Verwey medium, the final growth yield was generally even higher in the latter medium (Supplementary Fig. S1). After growth, the bacteria were either killed for 1 h at 56 °C or not. Subsequently, OMVs were isolated from equal amounts of bacterial cells, based on OD_600_, and analyzed by SDS-PAGE. For *B. pertussis*, OMV release was greatly enhanced by heat shock, and the highest amounts of OMVs appeared to be released by cells grown in Verwey medium (Fig. 1A). Heat shock also increased OMV release by *B. bronchiseptica*, but the influence of the medium was less in this case (Fig. 1B).

To quantify OMV release, we wished to determine the LPS and protein concentrations. However, starch, which is present in Verwey medium, appeared to interfere with the purpald assay for LPS quantification as well as with the BCA and, to a lesser extent, the Lowry assays for protein quantification. In addition, sOMV production was too low to quantify. To overcome these problems, we omitted starch from the Verwey medium and scaled-up OMV production. In our scaled-up setting, bacteria were grown in baffled flasks with an air:liquid ratio of 5:1, which has been reported to be critical for OMV production by *B. pertussis* (Gasperini et al., 2017). For *B. pertussis*, the media were also supplemented with 1 g/L of heptakis to enhance growth. After growth for two days, sOMVs and OMVs released by heat shock (hOMVs) were isolated. Also under these conditions, heat shock enhanced OMV production by both species, but the yield of hOMVs appeared rather independent of the growth medium used (Fig. 1C, D). Relative OMV production was quantified based on protein and LPS content by using the Lowry and purpald assay, respectively. Based on protein content, OMV release was ∼13-fold increased by *B. pertussis* grown in Verwey and SS medium after heat shock compared to sOMV production, whilst a ∼5-fold increase was measured after heat shock of cells grown in THIJS medium (Fig. 1E). However, based on LPS content, the increase in OMV release after heat shock was substantially lower and significant only for cells grown in Verwey medium, where a 3.5-fold increase was measured (Fig. 1F). Thus, the protein:LPS ratio appears to be increased in hOMVs relative to sOMVs. Based on protein content, heat shocking *B. bronchiseptica* grown in Verwey medium resulted in a ∼39-fold increase in OMV production and a ∼10-fold increase was observed after growth in SS and THIJS medium (Fig. 1G), whereas the increase based on LPS content was ∼18-fold, ∼13-fold and ∼11-fold, respectively (Fig. 1H). Taken together, heat shock drastically increases OMV release by both *Bordetella* species independent of growth medium.

### 3.2 Quality of OMVs induced by heat shock

**Fig. 2:**
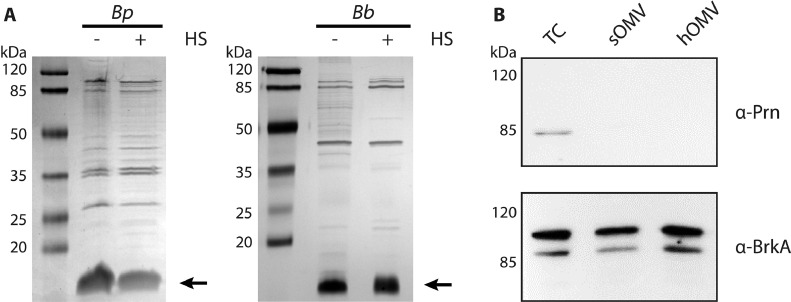
Comparison of protein content of OMV preparations. (**A**) SDS-PAGE analysis of OMVs from *B. pertussis* (*Bp,* left panel) and *B. bronchiseptica* (*Bb,* right panel) grown in Verwey medium isolated after heat shock (+ HS) or without heat shock (- HS). sOMVs were ∼10-fold (left panel) or ∼30-fold (right panel) concentrated compared to hOMVs. Proteins were stained with the Bradford reagent (left panel) or with the more sensitive silver stain (right panel). LPS (lipid A plus core moiety), which is also stained with both reagents, is indicated with an arrow at the right and molecular weight markers are shown at the left. (**B**) Western blot analysis of *B. pertussis* whole-cell (TC) lysates, sOMVs and hOMVs. sOMVs were 10-fold concentrated relative to hOMVs. Membranes were incubated with antibodies directed against pertactin (Prn) or BrkA. The two bands detected with the anti-BrkA antiserum presumably correspond to the full-length protein and the processed passenger domain of this autotransporter.

**Fig. 3:**
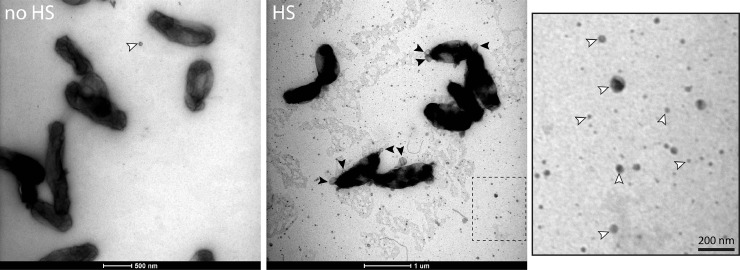
Morphology of *B. pertussis* cells and OMVs visualized by transmission electron microscopy. Bacterial cells were grown in Verwey medium and either exposed to heat shock for 1 h at 56 °C (HS) or not (no HS). The dashed box in the middle panel is ∼4-fold magnified and depicted in the right panel. Cell-associated OMVs (filled arrowheads) and released OMVs (open arrowheads) are indicated.

Application of a heat shock to the bacteria could affect the content or the conformation of the OMV proteins. To determine the quality of hOMVs, sOMVs of *B. pertussis* and *B. bronchiseptica* were ∼10- and 30-fold concentrated, respectively, via ultrafiltration, resulting in similar protein concentrations as in hOMVs. SDS-PAGE analysis showed very similar protein patterns in sOMV and hOMV preparations (Fig. 2A), indicating the absence of contamination of the hOMVs with other proteins, e.g. due to bacterial lysis. Consistently, transmission electron microscopy (TEM) confirmed the intactness of the bacterial cells after heat shock, although they appeared to be damaged as shown for *B. pertussis* in Fig. 3. These electron micrographs also confirmed the increased quantities of OMVs after heat shock (indicated by arrowheads in Fig. 3). Blebs which remained attached to the cell surface (black arrowheads) were bigger in size than released OMVs (open arrowheads). The diameters of released OMVs varied from 10 to 80 nm, with the majority of these OMVs being between 10 and 50 nm in diameter.

Important for the vaccine potential of OMVs is the presence of virulence factors. Therefore, the presence of two autotransporters, pertactin (Prn) and BrkA, in the OMVs was investigated by Western blotting. BrkA was equally detectable in sOMVs and hOMVs, but Prn, although present in whole-cell lysates, was not detectable in either OMV preparation (Fig. 2B). In any case, also in this respect, the heat shock does not seem to influence the quality of the OMVs isolated.

**Fig. 4:**
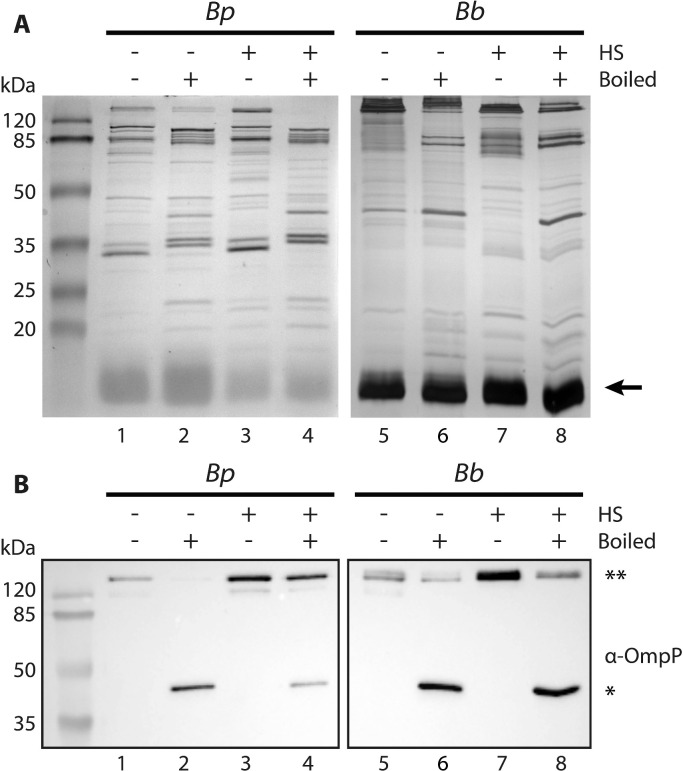
Heat modifiability of OMV proteins analyzed by semi-native SDS-PAGE. sOMVs (- HS) and hOMVs (+ HS) from bacteria grown in Verwey medium were either boiled or not in sample buffer before SDS-PAGE as indicated above the lanes. sOMVs of *B. pertussis* (*Bp*) and *B. bronchiseptica* (*Bb*) were ∼10-fold and ∼30-fold, respectively, concentrated compared to the hOMVs. (**A**) Proteins were stained with the Bradford reagent (left panel) or with the more sensitive silver stain (right panel). LPS (lipid A plus core moiety), which is also stained with both reagents, is indicated with an arrow at the right and molecular weight markers are shown at the left. (**B**) Western blot analysis of OMVs. Membranes were incubated with antisera directed against porin OmpP, which can be detected as a monomer (*) or as an oligomer (**).

Exposing the cells for 1 h to 56 °C may denature OMPs. To evaluate the effect of incubation at 56 °C on protein conformation on a macroscopic scale, the heat modifiability of OMPs in the OMV preparations was assessed by semi-native SDS-PAGE. OMPs are generally β-barrels that retain their native conformation when not heated in sample buffer before SDS-PAGE, and their heat denaturation results in a different electrophoretic mobility (Nakamura and Mizushima, 1976; Dekker et al., 1995; Grijpstra et al., 2013). OMV suspensions were mixed with sample buffer and either boiled or not before SDS-PAGE. The electrophoretic mobility of several OMPs appeared different in non-boiled samples compared to boiled samples (Fig. 4A, compare e.g. lane 1 vs. lane 2). These shifts were similar in OMVs isolated after heat shock (lane 3 vs. 4 and lane 7 vs. 8) or without heat shock (lane 1 vs. 2 and lane 5 vs. 6). Using Western blotting, we studied specifically the heat modifiability of porin OmpP, which forms oligomers in the OM (Armstrong et al., 1986). The monomeric form of OmpP was only detectable in boiled samples (Fig. 4B, lanes 2, 4, 6 and 8). These results suggest that the heat shock of the cells at 56 °C does not affect the conformation of the OMPs. Taken together, these data show that the protein quality is equal between sOMVs and hOMVs.

### 3.3 Effect of duration of heat shock on OMV release

**Fig. 5:**
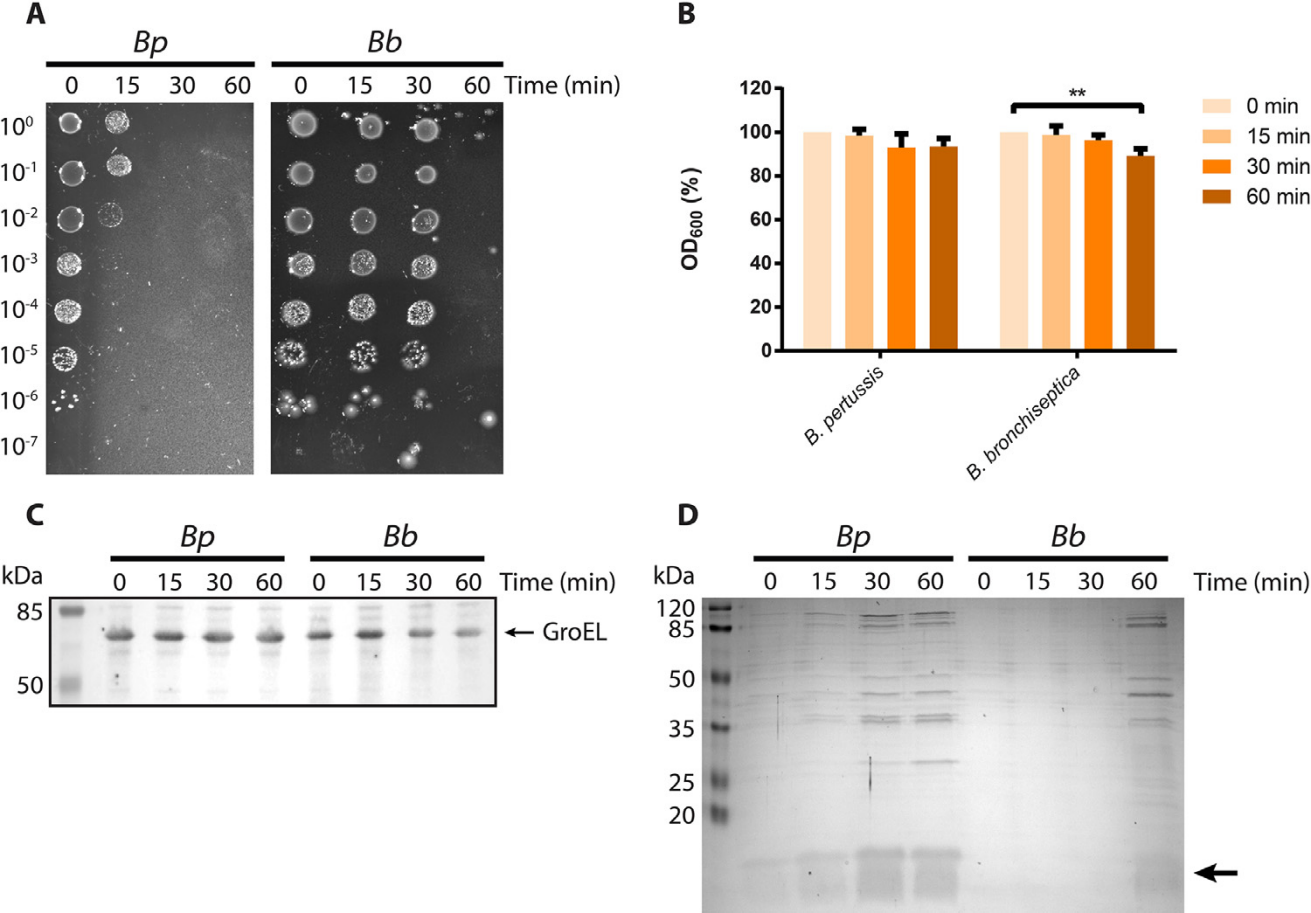
Influence of incubation period at 56 °C on cell viability, lysis and OMV release. *B. pertussis* (*Bp*) and *B. bronchiseptica* (*Bb*) cultures grown for one day in Verwey medium were incubated for the indicated time periods at 56 °C. (**A**) Viability of bacteria after incubation for different periods at 56 °C determined by plating 10-fold serial dilutions of cultures on BG plates. (**B**) OD_600_ of bacterial cultures expressed as percentage of the OD_600_ at *t* = 0. Mean values with standard deviations of three biological replicates are shown. Significant differences were determined using one-way ANOVA followed by Dunnett's multiple comparisons test using GraphPad Prism 6 and are indicated by asterisks (**, *p* ≤ 0.01). (**C**) Whole-cell lysates of bacterial cultures were analyzed by SDS-PAGE and Western blotting with an antiserum directed against GroEL. (**D**) SDS-PAGE analysis of OMVs isolated from the medium of bacterial cultures. OMVs were isolated from equal amounts of cells (based on OD_600_). LPS (lipid A plus core moiety), which is also stained with the Bradford reagent, is indicated with an arrow at the right and molecular weight markers are shown at the left.

Whilst incubation for 1 h at 56 °C greatly enhanced OMV release apparently without affecting protein content and conformation, we assessed the possibility of using shorter incubation periods to further reduce the risk of potential protein denaturation. Bacterial cultures were incubated for 0, 15, 30, or 60 min at 56 °C before OMV isolation. Plating of the *B. pertussis* cultures showed ∼10^4^-fold reduction in colony-forming units after 15 min of incubation at 56 °C (Fig. 5A). Complete killing was achieved after 30 min. *B. bronchiseptica* was more resistant to heat with complete killing being achieved only after 60 min. To assess if bacteria lysed at 56 °C, the OD_600_ was monitored. Even though incubation for 60 min was sufficient for effective killing of both species, lysis of the bacteria was hardly observed (Fig. 5B). This confirms the intactness of the heat-killed cells as suggested in the electron micrographs (Fig. 3). In *E. coli*, exposure to thermal stress induces expression of heat-shock proteins, such as GroEL (Gunasekera et al., 2008). To determine the response of *B. pertussis* and *B. bronchiseptica* to incubation at 56 °C, whole-cell lysates of bacterial cultures incubated for 0, 15, 30, or 60 min at 56 °C were analyzed by SDS-PAGE and Western blotting. GroEL was detected in the cells at all time points with no increase in production over time (Fig. 5C). Next, we determined the effect of incubation time on OMV release. SDS-PAGE analysis showed that OMV release by *B. pertussis* is enhanced after 15 min of incubation and maximal OMV release is reached after 30 min (Fig. 5D). In contrast, for *B. bronchiseptica* > 30 min of incubation was needed for efficient OMV release. This parallels the results of the viability assay, which indicated that *B. bronchiseptica* is more resistant to incubation at 56 °C.

### 3.4 Selective uploading of OMVs

**Fig. 6:**
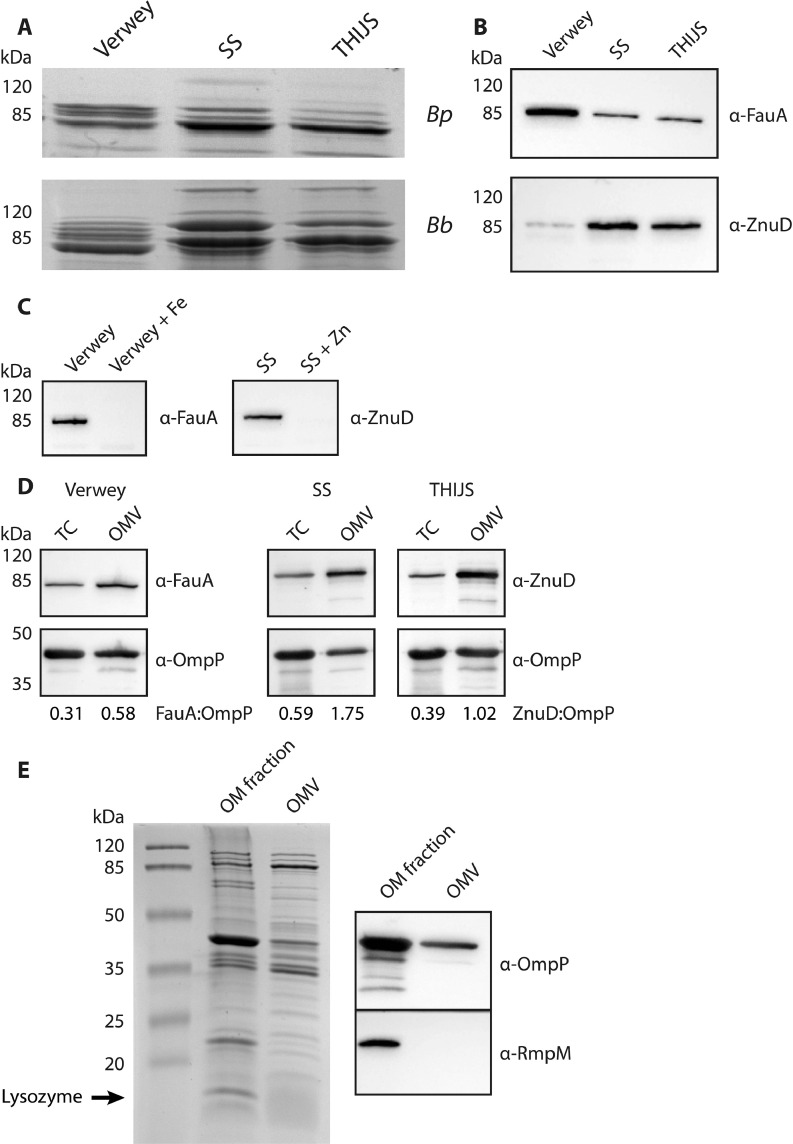
Preferential loading of OMPs into OMVs. (**A**) Zoomed-in ∼85-kDa range of the last three lanes of Fig. 1C (upper panel) and of Fig. 1D (lower panel). *Bp* = *B. pertussis, Bb* = *B. bronchiseptica*. (**B**) Western blot analysis of whole-cell lysates of *B. pertussis* grown in Verwey, SS or THIJS medium. Membranes were incubated with antisera directed against FauA or ZnuD as indicated. (**C**) Western blot analysis of whole-cell lysates of *B. bronchiseptica* grown in Verwey medium supplemented or not with 100 µM FeSO_4_•7H_2_O (left panel) or in SS medium supplemented or not with 1 µM ZnCl_2_ (right panel). Membranes were incubated with antisera directed against FauA or ZnuD as indicated. (**D**) Western blot analysis of whole-cell (TC) lysates and hOMVs of *B. pertussis* grown in Verwey, SS or THIJS medium. Membranes were incubated with antisera directed against porin OmpP, FauA or ZnuD. The ratio of the signals for FauA and ZnuD was determined relative to that of porin OmpP and is indicated. (**E**) A purified OM fraction (fraction 8 from Fig. S2A) and hOMVs of *B. pertussis* were analyzed by SDS-PAGE, and proteins were stained with the Bradford reagent (left panel) or blotted (right panel). Blots were incubated with antisera directed against OmpP or RmpM as indicated. Lysozyme, which is used during spheroplasting, is found in the OM fraction because it associates with the OM by electrostatic interactions. In all panels, the positions of molecular weight markers are shown at the left.

Dependent on the growth medium used, the protein patterns of the isolated OMVs showed differences, including in the ∼85-kDa range (Fig. 6A). As this molecular-weight range includes the TonB-dependent OM receptors, we hypothesized that these differences could be related to different availability of nutrient metals in the media. For example, in contrast to SS and THIJS medium, Verwey medium is not supplemented with a defined iron source, whilst another important nutrient metal, zinc, is not added to any of the media (Table S1). Nutrient-metal limitation leads to upregulation of metal acquisition systems, including TonB-dependent receptors. To investigate this possibility, the synthesis of FauA, the receptor of the *Bordetella* siderophore alcaligin (Brickman and Armstrong, 1999), and of BP3077, a homolog of the zinc receptor ZnuD of *N. meningitidis* (Stork et al., 2010) and henceforth also called ZnuD, was examined by Western blotting. For both *B. pertussis* and *B. bronchiseptica*, FauA was much more abundant after growth in Verwey medium (as shown for *B. pertussis* in Fig. 6B, upper panel), indicating that these cells are grown under iron limitation. Accordingly, siderophore production was also enhanced as detected by chrome azurol S assay (data not shown). Conversely, production of ZnuD was induced in SS and THIJS medium, indicating that these media are limiting for zinc (Fig. 6B, lower panel). Supplementation of Verwey medium with iron repressed FauA synthesis (Fig. 6C, left panel) and resulted in an increased final OD_600_ (data not shown). Similarly, ZnuD was repressed in SS medium supplemented with zinc (Fig. 6C, right panel), although no growth stimulation was observed (data not shown).

The most abundant OMPs in Gram-negative bacteria, including *Bordetella* spp., are usually the porins (Nikaido and Vaara, 1985; Armstrong et al., 1986). The dominant porin in *Bordetella* spp., called OmpP, has a molecular mass of 39.1 kDa and is expressed in both the Bvg^+^ and Bvg^−^ phase (Finn et al., 1995). Although a protein of ∼40 kDa was detected in both sOMVs and hOMVs, it was not a particularly prominent band. Several other proteins in the ∼85-kDa range, which includes the TonB-dependent receptors (see above), and in *B. pertussis* also in the ∼35-kDa range, probably corresponding to the C-terminal β-barrel domains of autotransporters such as BrkA, Prn and Tcf (Hamstra et al., 1995; Passerini de Rossi et al., 1999), were at least as abundant (Fig. 2A). This suggests that specific proteins are differentially uploaded into the OMVs and that the protein composition of OMVs does not necessarily reflect that of the bacterial OM. To investigate this possibility, whole-cell lysates and OMVs of *B. pertussis* were isolated after growth in Verwey, SS or THIJS medium, and differences in the ratio of the receptors FauA and ZnuD and that of porin OmpP were analyzed by Western blotting. The ratio of the signal for FauA relative to that for OmpP was 1.9-fold higher in OMVs than in whole cells grown in Verwey medium. Similarly, the ratio of the signal for ZnuD to that for OmpP was 3- and 2.6-fold higher in OMVs than in whole cells grown in SS and THIJS medium, respectively (Fig. 6D). These results indicate that, indeed, specific OMPs are selectively uploaded into OMVs.

To be able to directly compare the protein content of isolated OMVs and the bacterial OM, we wished to isolate a pure OM fraction. Therefore, the IM and OM were separated via isopycnic sucrose-gradient density centrifugation. Remarkably, whereas the bacterial OM usually has a higher buoyant density than the IM (Osborn et al., 1972), the IM markers FtsH and lactate dehydrogenase of *B. pertussis* were detected in fractions with a higher buoyant density than the OM markers porin OmpP and LPS (Fig. S2). We considered the possibility that this deviation was due to the 30-min incubation at 56 °C used to inactivate the bacteria. For safety reasons, this step was unfortunately unavoidable as, after spheroplast formation and freezing of the spheroplasts at −80 °C, viable cells were still detected in the suspension, which is a risk in the subsequent sonication step because of aerosol formation. Hence, we turned to *E. coli* to investigate the effect of heat inactivation on membrane separation. Heat inactivation did not affect the buoyant density of the OM, as can be concluded by the presence of the highest amounts of the OM markers, the porins OmpF/C and OmpA, in fractions 5 and 6 in either case (Fig. S3A). In contrast, the IM marker SecA was found, as expected, in lower density fractions if the cells were not heat inactivated, but it shifted to the high-density fractions after heat inactivation (Fig. S3B). In conclusion, although heat inactivation dramatically affects the buoyant density of the IM, we succeeded to separate IM and OM of *B. pertussis* and to obtain purified OM fractions.

After obtaining purified OM fractions, the total protein content of OMVs and OM could be compared. Relative to the proteins in the ∼85-kDa and ∼35-kDa ranges, the amounts of two major OMPs with apparent molecular weights of 40 kDa and 23 kDa were drastically decreased in OMVs compared to the OM fraction (Fig. 6E, left panel). Western blotting confirmed the identity of the 40-kDa band as porin OmpP, whereas the 23-kDa band could be identified as BP0943, a homolog of reduction-modifiable protein M (RmpM) of *N. meningitidis* (Grizot and Buchanan, 2004) and hereafter also called RmpM (Fig. 6E, right panel). Together, these results demonstrate that the protein content of OMVs deviates from that of the OM.

## 4 Discussion

Because of the resurgence of pertussis cases in the last 20 years, the demand for a third-generation vaccine is rising. This vaccine could be OMV based, but OMV production by *Bordetella* species is relatively low. The first report of *B. pertussis* OMVs dates back to 1970 (Morse and Morse, 1970). Since then, different methods have been described to isolate OMVs from *B. pertussis*. These include sonication of bacterial cells and the use of detergents (Hozbor et al., 1999), but these harsh treatments could alter OMV properties important for vaccine purposes. Recently, an optimized protocol to isolate sOMVs from cell-free supernatant using ultracentrifugation, retaining native OMV properties, was reported (Gasperini et al., 2017). In this study, we have demonstrated that incubation of *B. pertussis* and *B. bronchiseptica* at 56 °C drastically enhances OMV release. In our scaled-up setting, efficient OMV release by heat shock was independent of the growth medium used.

Released OMVs were quantified based on protein and LPS content. The increase in OMV release by *B. pertussis* upon heat shock appeared lower when it was quantified based on LPS rather than on protein content (Fig. 1E, F). This suggests that the protein:LPS ratio is higher in hOMVs compared to sOMVs. This higher protein:LPS ratio in hOMVs, which was also observed in the SDS-PAGE gels (e.g. see Fig. 2A, left panel), may be advantageous for vaccine development as the lower LPS content may result in lower reactogenicity of the vaccine. The protein:LPS ratio did not seem to differ between sOMVs and hOMVs of *B. bronchiseptica*. The LPS structures of *B. pertussis* and *B. bronchiseptica* differ in several aspects. For example, in contrast to *B. pertussis* LPS, *B. bronchiseptica* LPS contains an O-antigen, and, also, differences in the acylation pattern of the lipid A moiety of the LPS have been reported (MacArthur et al., 2007). Possibly, the LPS of *B. pertussis* is more easily released from the OM into the environment upon heat shock.

Several studies have shown the influence of growth temperature on OMV production in other bacteria. In *E. coli*, vesiculation is ∼5-fold increased when the bacteria are grown at 37 °C compared to 30 °C (McBroom and Kuehn, 2007). This effect is strongly enhanced in a *degP* mutant with more than 150-fold increase in OMV release compared to the wild-type strain grown at 37 °C. It was hypothesized that this is due to elevated levels of misfolded proteins in the periplasm of *degP* mutants and that the bacteria relieve the resulting stress by hypervesiculation. In contrast, OMV production by *Pseudomonas aeruginosa* was not affected when cultures were shifted from 25 °C to 37 °C or 39 °C (MacDonald and Kuehn, 2013). We showed that the effect of temperature stress on OMV release is already detectable after 15 and 60 min in static conditions for *B. pertussis* and *B. bronchiseptica*, respectively. However, GroEL levels did not increase after the temperature shift, presumably because protein synthesis is immediately and completely inhibited at 56 °C. This would indicate that the increased OMV release is not an active response of the bacterial cells to the heat shock but a biophysical membrane process resulting in increased blebbing, however without significant cell lysis. Previously, severe heat stress (30 min at 55 °C) has been reported to result in cell death with concomitant OM blebbing in *E. coli* (Katsui et al., 1982).

Characterization of sOMVs and hOMVs indicated that their quality with respect to protein composition is comparable. Although differences in lipid composition were detected, most notably a higher content of lysophospholipids in hOMVs, this difference did apparently not influence their immunogenic properties when tested in vitro by stimulation of porcine bone-marrow-derived macrophages (Balhuizen et al., 2020; see accompanying paper). Importantly, virulence factor BrkA was detectable in both OMV preparations. BrkA is a relevant vaccine candidate as shown by enhanced bacterial clearance by mice immunized with an aP vaccine containing pertussis toxin and filamentous hemagglutinin if this vaccine was supplemented with BrkA (Marr et al., 2008). A proteomic study on *B. pertussis* OMVs showed that ∼14% of total proteins in OMVs from Bvg^+^-phase bacteria is BrkA (Gasperini et al., 2018). In another study, BrkA and Vag8 were the major proteins in a *B. pertussis* OMV formulation comprising 21% and 50%, respectively, of the total OMV protein content (Kanojia et al., 2018). On the other hand, only 1.4% of total OMV protein content was Prn according to the latter study. We were not able to detect Prn in either of the OMV preparations, possibly because its abundance is too low.

TEM analysis showed that the majority of isolated OMVs from *B. pertussis* had a diameter between 10 and 50 nm, which is in accordance with a size of 20–40 nm as measured by dynamic light scattering for *B. bronchiseptica* OMVs (Balhuizen et al., 2020; see accompanying paper). This is relatively small, considering the range of OMV diameters reported in the literature, and smaller than the sOMVs analyzed by Gasperini et al., which ranged between 70 and 230 nm according to nanoparticle tracking analysis (NTA) (Gasperini et al., 2017). However, this might reflect a limitation of the NTA method in detecting small-size particles as the electron micrographs in the latter study showed OMVs with a much smaller diameter, i.e. in the 10–50 nm range, which is similar to those in our study. OMVs isolated from cell-free supernatant of *B. pertussis* in the presence of glutaraldehyde ranged from 150 to 250 nm (Hozbor et al., 1999). A study in *E. coli* showed that the diameter of isolated OMVs was smaller than 50 nm (Daleke-Schermerhorn et al., 2014). Apparently, the OMV diameter varies not only between species but it also depends on the isolation method.

OMVs isolated from bacteria grown in various media showed variation in protein patterns, which appears to be due, at least in part, to differences in nutrient-metal availability. The synthesis of the alcaligin receptor FauA was induced in bacteria grown in Verwey medium, indicating that this medium is limiting for iron. Indeed, in contrast to SS and THIJS media, which contain iron sulfate, no defined iron source is added to the Verwey medium. In contrast to Verwey medium, SS and THIJS medium are limiting for zinc, resulting in higher production of the zinc receptor ZnuD. Neither of the media is supplemented with a zinc source, but, probably, one of the constituents of the Verwey medium, e.g. casamino acids, contains sufficient amounts of zinc as a contaminant to repress ZnuD synthesis. Furthermore, we have shown that FauA and ZnuD are enriched in OMVs relative to porin suggesting that *B. pertussis* OMVs may play a role in metal acquisition. In several Gram-negative bacteria, e.g. *N. meningitidis* and *Porphyromonas gingivalis*, proteins involved in metal acquisition are enriched in OMVs compared to the OM (Lappann et al., 2013; Veith et al., 2014), suggesting involvement of OMVs in metal acquisition. Accordingly, it has been shown in *Mycobacterium tuberculosis* (Prados-Rosales et al., 2014), *P. aeruginosa* (Lin et al., 2017), and also in *B. pertussis* (Gasperini et al., 2017), that (O)MVs are able to bind iron and transport it to bacterial cells. Apart from the role of these metal receptors in the bacterium itself, these receptors have vaccine potential. Studies have shown that iron receptors in several Gram-negative bacteria (Afonina et al., 2006; Alteri et al., 2009; Hu et al., 2012) and ZnuD in *N. meningitidis* (Stork et al., 2010; Hubert et al., 2013) are important vaccine candidates. Advantageously, we have shown that FauA and ZnuD are already produced by *B. pertussis* and *B. bronchiseptica* in media that are commonly used to grow these bacteria.

To be able to compare the protein content of OMVs and the native OM, we wished to separate the IM and OM using sucrose gradient density centrifugation, which is notoriously difficult in *B. pertussis* (see, e.g., Ezzell et al., 1981; Jacob-Dubuisson et al., 1999). However, for the first time to our knowledge, we succeeded to separate IM and OM from *B. pertussis* in this way and to obtain a purified OM fraction. The IM had a higher buoyant density than the OM, which, as we demonstrated, is probably an artifact due to the inactivation of cells by heat. Comparison of the protein content of a purified OM fraction with OMVs showed great similarities. However, the relative abundance of two major OMPs appeared to be very low in OMVs. One of them was porin OmpP. The other one could be identified as a homolog of the *N. meningitidis* protein RmpM. RmpM has an OmpA_C-like domain at the C terminus, which mediates non-covalent binding to PG (Koebnik, 1995; Grizot and Buchanan, 2004). Presumably via its N-terminal domain, RmpM is also firmly associated with porins in the OM (Jansen et al., 2000) and thus mediates the attachment of the OM to the PG layer. Accordingly, deletion of *rmpM* appeared to impair the interaction between the OM and the PG layer in *N. meningitidis* (Steeghs et al., 2002). Assuming a similar function of RmpM in *Bordetella* and based on the low RmpM content of the OMVs, we hypothesize that OMVs emerge at specific OM domains with a low RmpM content. As RmpM associates with porins in the OM, the OMVs generated will also have a low porin content. The low abundance of RmpM in OMVs may be beneficial for further vaccine-development purposes since it has been shown that *N. meningitidis* RmpM induces blocking antibodies, which inhibit complement-mediated bacterial killing (Munkley et al., 1991). A more extensive comparison of the protein content of OMVs and purified OMs by proteomic analysis will be done in future experiments.

In conclusion, we have shown that heat inactivation of *Bordetella* spp. stimulates the release of OMVs. With respect to protein content, the resulting hOMVs do not deviate from those of sOMVs. Therefore, applying a heat shock appears to be a suitable method for the commercially viable development of novel OMV-based vaccines.

## Funding

This work was supported by a TTW Perspectief grant (14921 and 14924) of The Netherlands Organization for Scientific Research (NWO) with financial aid from GlaxoSmithKline Biologicals SA and PULIKE Biological Engineering Inc.

## CRediT authorship contribution statement

**Eline F. de Jonge:** Conceptualization, Investigation, Writing - original draft. **Melanie D. Balhuizen:** Investigation, Writing - review & editing. **Ria van Boxtel:** Investigation. **Jianjun Wu:** Investigation. **Henk P. Haagsman:** Supervision, Writing - review & editing. **Jan Tommassen:** Conceptualization, Supervision, Funding acquisition, Writing - original draft.

## Declaration of Competing Interest

Part of this work is included in a European patent application (EP20187477.3) with EFdJ, MDB, HPH, and JT as inventors. The authors declare that they have no known competing financial interests or personal relationships that could have appeared to influence the work reported in this paper.

## Appendix Supplementary materials

Image, application 1
