# Supplementary material for: Heat shock enhances outer-membrane vesicle release in Bordetella spp
Source: Curr Res Microb Sci. 2020 Sep 17;2:100009. doi: 10.1016/j.crmicr.2020.100009 (PMC8610307; doi:10.1016/j.crmicr.2020.100009)
Supplement: Supplementary file 1 [file mmc1.docx]

**Table S1.** Composition of Verwey (A), Stainer-Scholte (B) and THIJS (C) medium.

**A.** Verwey medium (pH 7.4)

| Component | Amount per liter |
| --- | --- |
| Starch from potato (Sigma-Aldrich) | 1.0 g |
| KCl | 0.2 g |
| KH_2_PO_4_ | 0.5 g |
| MgCl_2_·6H_2_O | 0.1 g |
| Nicotinic acid | 0.02 g |
| Casamino acids (Bacto) | 14.0 g |
| L-Glutathione reduced | 0.01 g |

**B.** Stainer-Scholte medium

*Basal medium (pH 7.6)*

| Component | Amount per liter |
| --- | --- |
| L-Glutamic acid monosodium salt | 10.72 g |
| L-Proline | 0.24 g |
| NaCl | 2.5 g |
| KH_2_PO_4_ | 0.5 g |
| KCl | 0.2 g |
| MgCl_2_·6H_2_O | 0.1 g |
| CaCl_2_·2H_2_O | 0.02 g |
| Tris base | 1.52 g |
| Stainer-Scholte supplement | 10 mL |

*Supplement (100x)*

| Component | Amount per liter |
| --- | --- |
| L-Cystine (dissolved in 4 M HCl) | 4.0 g |
| FeSO_4_·7H_2_O | 1.0 g |
| Nicotinic acid | 0.4 g |
| L-Ascorbic acid | 2.0 g |
| L-Glutathione reduced | 10.0 g |

**C.** THIJS medium

*Basal medium (pH 7.4)*

| Component | Amount per liter |
| --- | --- |
| NaCl | 3.32 g |
| NH_4_Cl | 0.11 g |
| KH_2_PO_4_ | 0.5 g |
| KCl | 0.5 g |
| MgCl_2_·6H_2_O | 0.1 g |
| Tris base | 1.53 g |
| L-Glutamic acid monosodium salt | 1.87 g |
| L-Lactate (40% w/v) | 3.76 mL |
| 5 M NaOH | 2.071 mL |
| THIJS supplement | 10 mL |

*Supplement (100x)*

| Component | Amount per liter |
| --- | --- |
| L-Cystine (dissolved in 4 M HCl) | 4.0 g |
| FeSO_4_·7H_2_O | 1.0 g |
| Nicotinic acid | 0.4 g |
| L-Ascorbic acid | 2.0 g |
| L-Glutathione reduced | 10.0 g |
| CaCl_2_·2H_2_O | 2.6 g |

Table S2. Primers used in this study

| Name | Sequence^a^ | Restriction site |
| --- | --- | --- |
| BP0840_Fw | GATCATATGGAAACGTCGGTCACCCTGTA | NdeI |
| BP0840_Rev | GATGGATCCTTAGAAGCGGTGACGGATAC | BamHI |
| FauA_Fw | GATCATATGCAGGAAGCGCGAACGGGG | NdeI |
| FauA_Rev | GATGGATCCTCAATACTGCGCCCGCAAGT | BamHI |
| ZnuD_Fw | GATCATATGCAGGCAAGCGCGGACGGTAC | NdeI |
| ZnuD_Rev | GATGGATCCTCAGTACGTCAGCCGCACGC | BamHI |

^a^ Recognition sites for the restriction enzymes indicated in the last column are underlined.


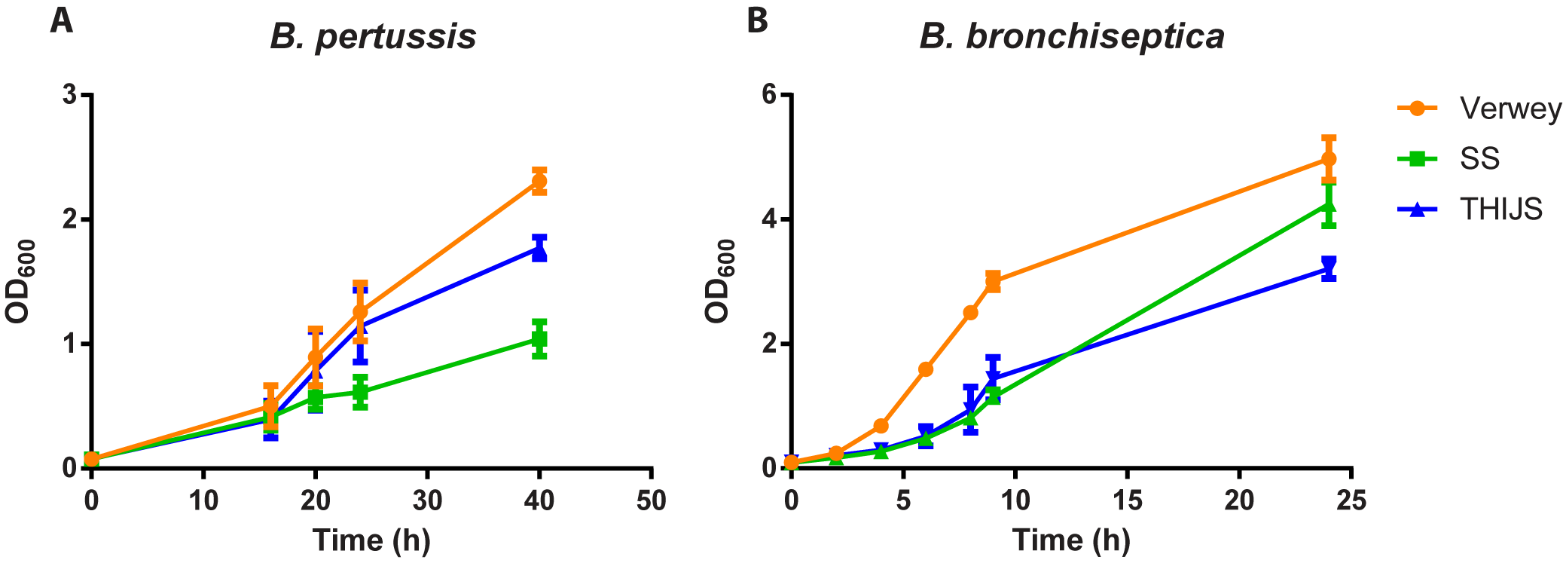


**Fig. S1.** Growth curves of *B. pertussis* and *B. bronchiseptica* in different media. Growth curves of *B. pertussis* (**A**) and *B. bronchiseptica* (**B**) measured as OD_600_ in three different media as indicated. For *B. pertussis*, the media were supplemented with heptakis to adsorb free fatty acids that inhibit its growth (Frohlich et al., 1996). Graphs show mean values with standard deviations of three biological replicates.


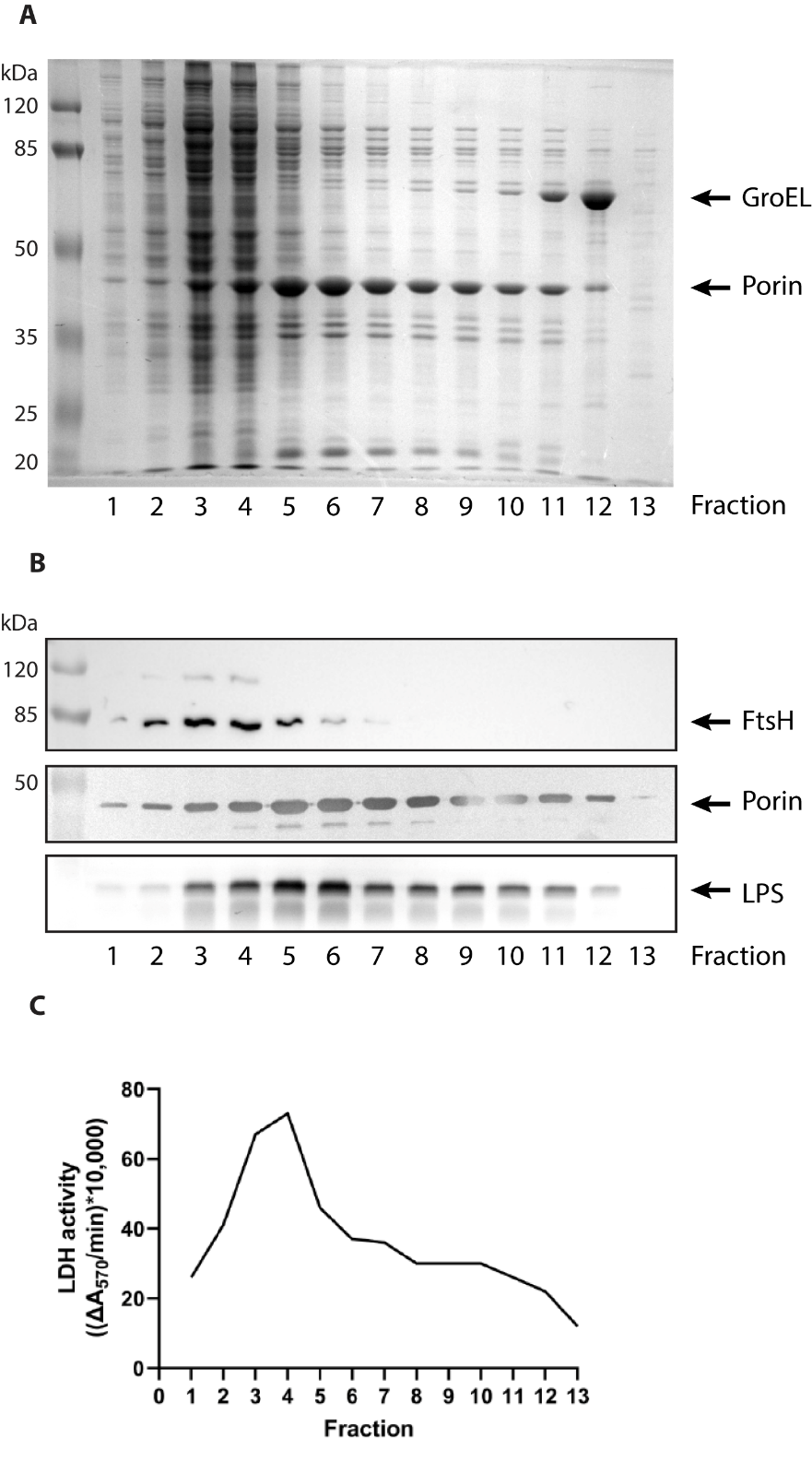

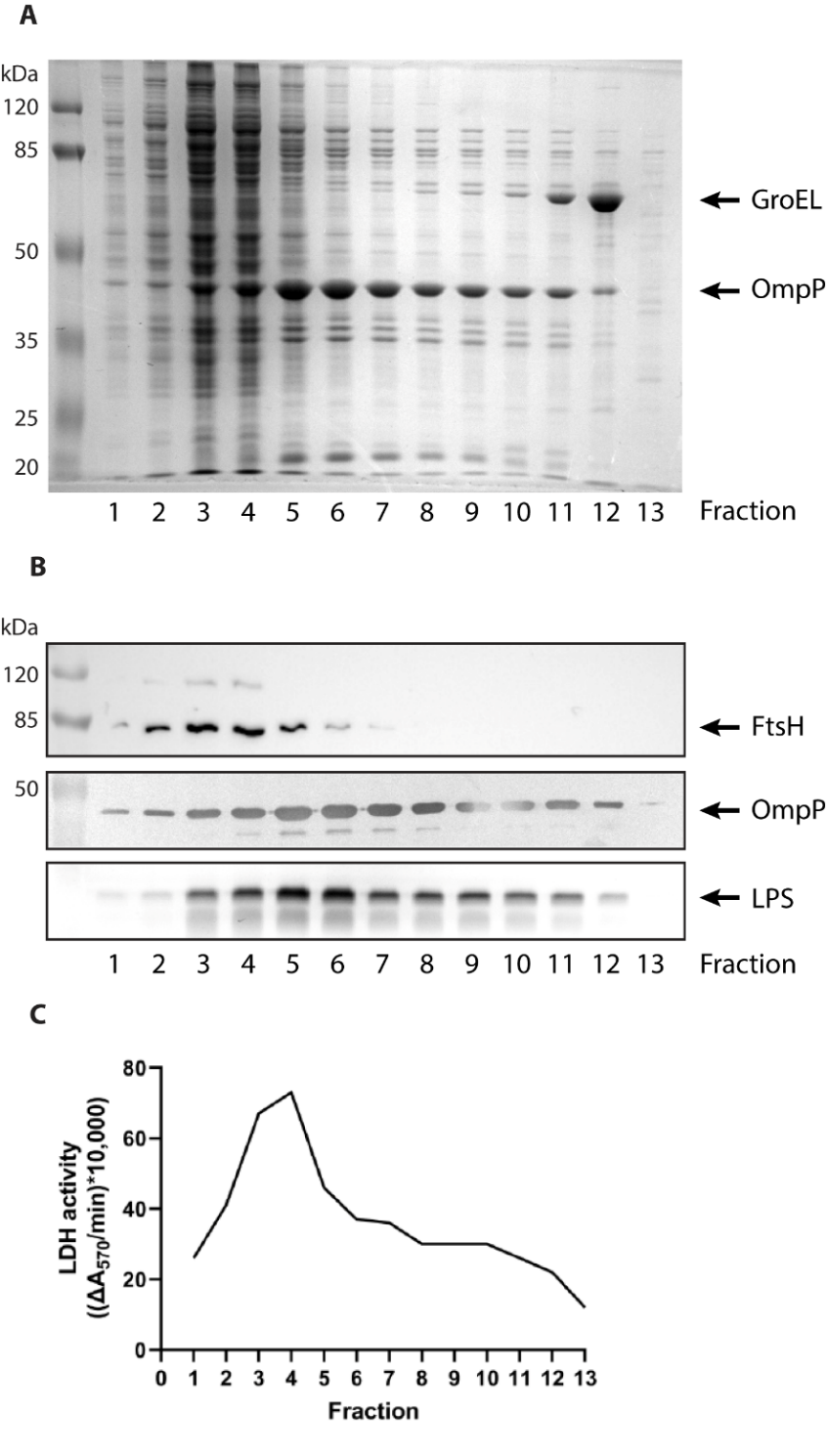


**Fig. S2.** Separation of *B. pertussis* IM and OM. Spheroplasts of heat-inactivated *B. pertussis* were disrupted by sonication, and the isolated membrane fraction was loaded onto a sucrose gradient. After centrifugation, the sucrose gradient was fractionated with fraction 1 being at the bottom of the tube. (**A**) Isolated fractions were analyzed by SDS-PAGE and stained with the Bradford reagent. Two prominent proteins, GroEL and porin OmpP, are indicated. Molecular weight markers are shown at the left. (**B**) Fractions were analyzed by Western blotting using antisera directed against IM marker FtsH or OM marker OmpP, or by SDS-PAGE and stained with silver to visualize OM marker LPS. Molecular weight markers are shown at the left. (**C**) Lactate dehydrogenase (LDH) activity in the different fractions. We remark that the total LDH activity is low due to partial loss of enzyme activity during heat inactivation of the cells.


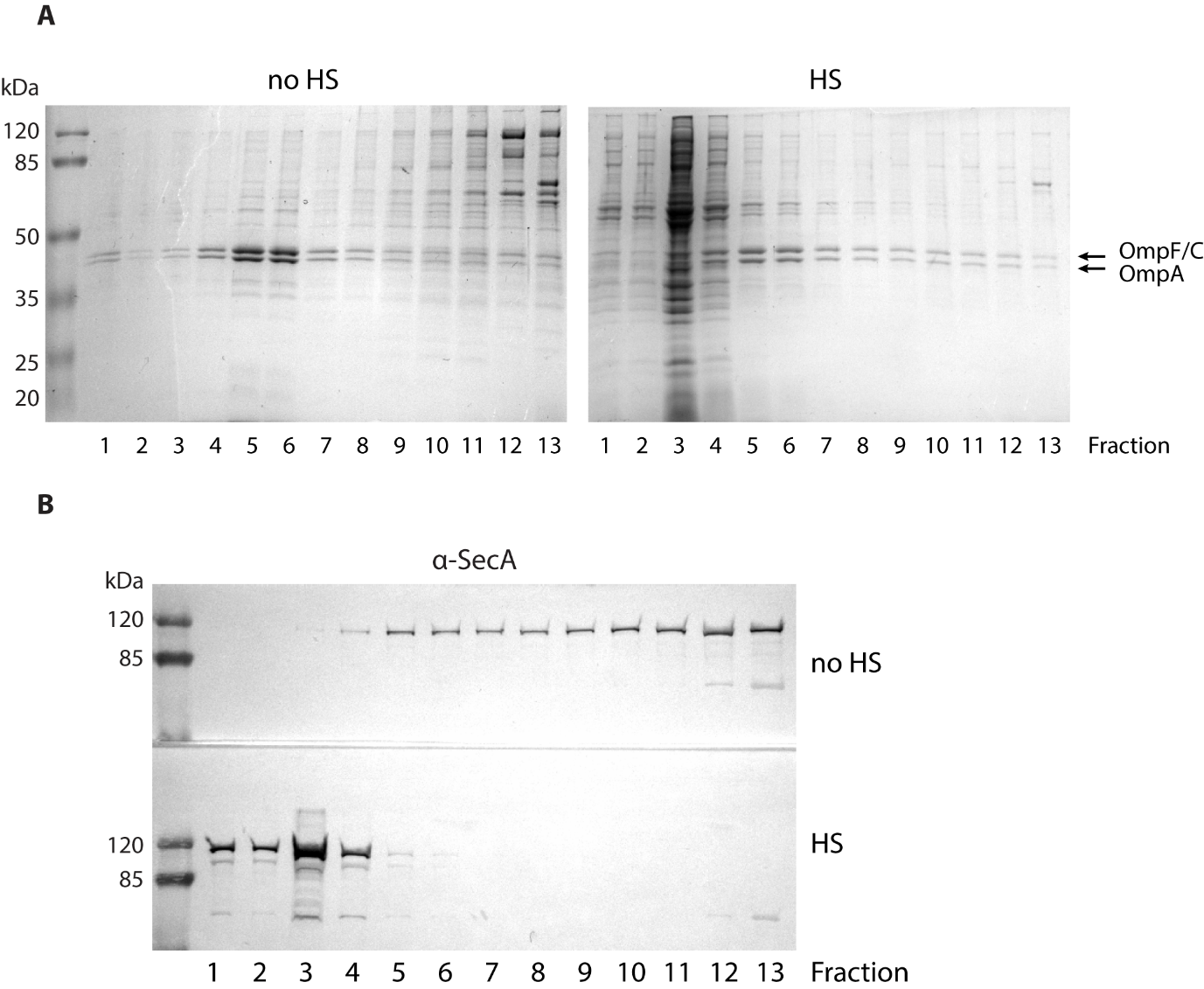


**Fig. S3.** Separation of *E. coli* IM and OM. *E. coli* cells were either inactivated by incubation for 30 min at 56℃ or not before converting them to spheroplasts. The spheroplasts were disrupted by sonication, and the isolated membranes were loaded onto a sucrose gradient. After centrifugation, the sucrose gradient was fractionated with fraction 1 being at the bottom of the tube. (**A**) Isolated gradient fractions of cells that were either not inactivated by heat (no HS) or heat inactivated (HS) were analyzed by SDS-PAGE and stained with the Bradford reagent. The positions of the OM markers OmpF/C and OmpA are indicated at the right. (**B**) Fractions were analyzed by Western blotting using an antiserum directed against IM marker SecA. In both panels, the positions of molecular weight markers are shown at the left.
